# Association between systemic hemodynamics and septic acute kidney injury in critically ill patients: a retrospective observational study

**DOI:** 10.1186/cc13133

**Published:** 2013-11-29

**Authors:** Matthieu Legrand, Claire Dupuis, Christelle Simon, Etienne Gayat, Joaquim Mateo, Anne-Claire Lukaszewicz, Didier Payen

**Affiliations:** 1 Department of Anesthesiology and Critical Care, Lariboisière Hospital, Assistance Publique-Hôpitaux de Paris, University of Paris 7 Denis Diderot, 2 rue Ambroise-Paré, 75475 Paris, Cedex 10, France; 2 University of Paris Diderot, EA-3509, F-75475 Paris, France; 3 INSERM U717, Clinical Epidemiology and Biostatistics, Saint-Louis Hospital, 1 Avenue Claude Vellefaux, 75010, Paris, France; 4 INSERM UMR 1160, Alloimmunity, Autoimmunity, Transplantation, Saint-Louis Hospital, 1 Avenue Claude Vellefaux, 75010, Paris, France

## Abstract

**Introduction:**

The role of systemic hemodynamics in the pathogenesis of septic acute kidney injury (AKI) has received little attention. The purpose of this study was to investigate the association between systemic hemodynamics and new or persistent of AKI in severe sepsis.

**Methods:**

A retrospective study between 2006 and 2010 was performed in a surgical ICU in a teaching hospital. AKI was defined as development (new AKI) or persistent AKI during the five days following admission based on the Acute Kidney Injury Network (AKIN) criteria. We studied the association between the following hemodynamic targets within 24 hours of admission and AKI: central venous pressure (CVP), cardiac output (CO), mean arterial pressure (MAP), diastolic arterial pressure (DAP), central venous oxygen saturation (ScvO_2_) or mixed venous oxygen saturation (SvO_2_).

**Results:**

This study included 137 ICU septic patients. Of these, 69 had new or persistent AKI. AKI patients had a higher Simplified Acute Physiology Score (SAPS II) (57 (46 to 67) vs. 45 (33 to 52), *P* < 0.001) and higher mortality (38% vs. 15%, *P* = 0.003) than those with no AKI or improving AKI. MAP, ScvO_2_ and CO were not significantly different between groups. Patients with AKI had lower DAP and higher CVP (*P* = 0.0003). The CVP value was associated with the risk of developing new or persistent AKI even after adjustment for fluid balance and positive end-expiratory pressure (PEEP) level (OR = 1.22 (1.08 to 1.39), *P* = 0.002). A linear relationship between CVP and the risk of new or persistent AKI was observed.

**Conclusions:**

We observed no association between most systemic hemodynamic parameters and AKI in septic patients. Association between elevated CVP and AKI suggests a role of venous congestion in the development of AKI. The paradigm that targeting high CVP may reduce occurrence of AKI should probably be revised. Furthermore, DAP should be considered as a potential important hemodynamic target for the kidney.

## Introduction

Sepsis is the leading cause of acute kidney injury (AKI) in critically ill patients [1] and is associated with high in-hospital mortality, exceeding 50% when AKI is part of a multiple organ failure syndrome. Among the factors predisposing patients to AKI, two seem to predominate: (1) macro- and microhemodynamic impairment [2] and (2) immune toxicity in kidney tissue cells [3]. Although it has been suggested for a long time, the role of compromised systemic hemodynamics leading to AKI in septic patients remains a source of debate [4,5]. Surprisingly, the association between the development of AKI and systemic hemodynamic parameters has not been extensively investigated [6], particularly with regard to venous congestion. Although studies have been done in which renal venous congestion in heart failure [7,8] or experimental septic AKI was suggested, renal congestion has not been explored in septic patients [9]. The present retrospective study was designed to investigate the relation between systemic hemodynamics and the progression of AKI in severe sepsis or septic shock patients.

## Materials and methods

### Patients

**Figure 1:**
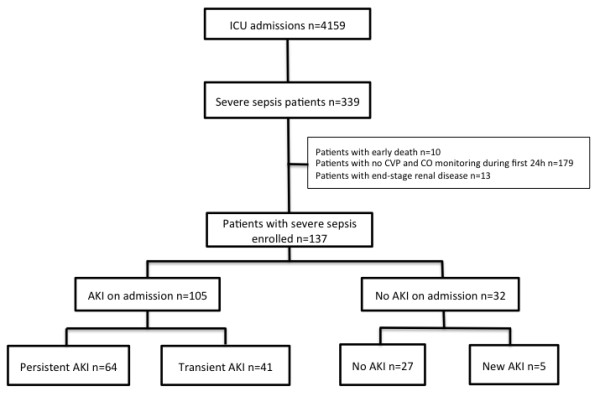
**Flowchart of patients included in the study.** AKI, acute kidney injury. CO, cardiac output; CVP, central venous pressure.

This study was approved by our local ethics committee (Ethics Committee for the Evaluation of Biomedical Research Projects of North Paris), and the need for informed consent was waived because of the noninterventional, retrospective design of the study. Figure 1 shows the flowchart of the study. All patients with a diagnosis of severe sepsis or septic shock [10] admitted to our surgical ICU between January 2006 and December 2010 were screened. Patients were excluded if they did not have central venous pressure (CVP) and/or cardiac output (CO) monitoring (for example, cervical cellulitis) or if they died within 24 hours of admission. The selected cohort was treated according to our local institution recommendations: antibiotics were administered within the 6 hours after admission and fluid resuscitation was initiated with crystalloids (NaCl 0.9% or Ringer’s lactate solution) or with gelatin solution for some others (Plasmion; Fresenius Kabi France, Sèvres, France). Norepinephrine was largely the most frequently used vasopressor, and some patients were given a combination of norepinephrine and either epinephrine or dobutamine in the presence of cardiac dysfunction, a decision made by the physician in charge. When mechanical ventilation was initiated, tidal volume was set to maintain an inspiratory plateau pressure less than 30 cmH_2_O.

### Data collection

The patients’ characteristics are presented in Table 1. The physiological parameters are presented in Table 2. The following hemodynamic parameters were collected during the first 24 hours after admission: CVP, CO, systolic arterial pressure, diastolic arterial blood pressure (DAP), mean arterial blood pressure (MAP), central venous oxygenation saturation (ScvO_2_) and mixed oxygen venous saturation (SvO_2_). CO was measured with a pulmonary artery catheter and a Vigilance II monitor (Edwards Lifesciences, Irvine, CA, USA) or a transesophageal Doppler monitor (CardioQ-ODM; Deltex Medical, Chichester, UK). Because of hemodynamic variations during the unstable initial phase, the lower limits and the upper limits of the range (ULR) and the mean value over the first 24 hours were recorded. The association between the hemodynamic targets within 24 hours from admission (CVP = 8 to 12 mmHg; CO >5 L/min, MAP >65 mmHg, DAP >50 mmHg, ScvO_2_ >70% and SvO_2_ >60% [11,12]) and progression or development of AKI was investigated. The ULR of each target was defined as the highest value achieved at any time within the first 24 hours after admission, and the lower limit range was defined as the lowest value of the hemodynamic parameter during the whole 24-hour period.

**Table 1:** **Patient characteristics**^**a**^

**Characteristics**	**All patients (*N*** **= 137)**	**AKI − (*N*** **= 68)**	**AKI + (*N*** **= 69)**	***P *** **value**
Age (years)	71.1 (56.3 to 79.8)	68.5 (49.8 to 77.5)	73.4 (60.3 to 80.7)	0.044
Males	60 (45)	35 (41.5)	42 (60.9)	0.38
Comorbidities				
COPD	12 (9)	9 (13)	3 (4)	0.06
Diabetes mellitus	20 (15)	7 (10)	13 (19)	0.16
Hypertension	59 (43)	23 (34)	36 (52)	0.03
Heart failure	19 (14)	7 (10)	12 (17)	0.23
CAD	16 (12)	6 (9)	10 (14)	0.3
Liver disease	10 (7)	4 (6)	6 (9)	0.53
Cancer	39 (28)	19 (30)	20 (29)	0.89
Medication before admission				
NSAIDs	7 (5)	3 (4)	4 (6)	0.71
Diuretics	28 (20)	11 (16)	17 (25)	0.22
Statins	24 (17)	9 (13)	15 (22)	0.19
Steroids	9 (7)	3 (4)	6 (9)	0.32
β-blockers	29 (2)	12 (18)	17 (25)	0.32
Antiplatelet therapy	27 (20)	13 (19)	14 (10)	0.86
Organ failure				
Mechanical ventilation	118 (86)	55 (81)	63 (91)	0.08
SAPS II	50 (39 to 60)	45 (33 to 52)	57 (46 to 67)	<0.0001
Norepinephrine	131 (96)	65 (95)	66 (95)	0.82
Epinephrine	17 (12)	4 (6)	13 (19)	0.02
Dobutamine	4 (3)	3 (4)	1 (1)	0.3
Dose of norepinephrine^b^	0.44 (0.20 to 0.73)	0.31 (0.16 to 0.54)	0.56 (0.30 to 0.95)	0.0005
Dose of epinephrine^b^	0.31 (0.14 to 0.40)	0.23 (0.15 to 0.32)	0.31 (0.12 to 42)	0.70
Dose of dobutamine^b^	5 (5 to 5)	5 (5 to 5)	5 (5 to 5)	1
Hydrocortisone	19 (14)	2 (10.5)	17 (25.4)	0.17
Lactate (mmol/L)	2.8 (1.9 to 4.8)	2.7 (1.8 to 3.9)	3 (2.1 to 6.2)	0.06
Serum creatinine (μmol/L)	141.5 (83 to 215.8)	88 (68 to 143.2)	185 (134 to 255)	<0.0001
Bilirubin (mg/ml)	16 (9 to 27.8)	14 (8 to 21.8)	16 (11 to 33)	0.04
Platelet count (g/ml)	65 (30.8 to 98.2)	63 (28.8 to 89.2)	67 (31 to 102)	0.45
Hemoglobin (g/dl)	10.3 (9.4 to 12)	10.3 (9.9 to 12)	10.4 (9.3 to 12.1)	0.39
Base deficit (mmol/L)	−7.7 (−11.3 to −3.4)	−5 (−8.9 to −2.3)	−8.8 (−13 to −5.8)	0.0006
Fluid balance (ml)	3,480 (1,945 to 5,351)	2,905 (1,350 to 4,717.5)	3,591.5 (2,597.5 to 5,714)	0.008
Origin of sepsis				
Abdomen	78 (57)	37 (54)	41 (59)	0.55
Lung	28 (20)	16 (23)	12 (17)	0.37
Urinary tract	9 (7)	3 (4)	6 (9)	0.31
Soft tissue	8 (6)	5 (7)	3 (4)	0.45
Other	20 (15)	11 (16)	9 (13)	0.6
Nephrotoxic agents				
Contrast media	82 (61)	41 (60)	41 (60)	0.21
Vancomycin	53 (39)	29 (43)	24 (35)	0.31
Aminoglycosides	9 (7)	5 (7)	4 (6)	0.71
Colloids	108 (79)	47 (69)	61 (88)	0.005

^a^AKI−, no or transient acute kidney injury; AKI+, new or persistent acute kidney injury; CAD, Coronary artery disease; COPD, Chronic obstructive pulmonary disease; NSAID, Nonsteroidal anti-inflammatory drug; SAPS II, Simplified Acute Physiology Score II. ^b^Data are micrograms per kilogram per minute among patients receiving the drug. The data in the table are expressed as median (interquartile range) or number (%).

**Table 2:** **Hemodynamic parameters during the first 24 hours after admission**^**a**^

**Parameters**	**AKI − (*N*** **= 68)**	**AKI + (*N*** **= 69)**	***P *** **value**
CO (mean)	4.6 (3.6 to 6.2)	4.9 (3.8 to 6.7)	0.41
CO (LLR)	3.7 (3 to 5.4)	3.8 (2.9 to 4.8)	0.76
CO (ULR)	5.7 (3.9 to 7.1)	6 (5.1 to 8.1)	0.14
ScvO_2_ (mean)	74.5 (71.7 to 78.4)	74.5 (67.3 to 77.5)	0.26
SvcO_2_ (LLR)	71 (65 to 75)	67 (60.1 to 72.3)	0.058
SvcO_2_ (ULR)	80 (76 to 84.2)	80 (75.6 to 84)	0.92
SAP (mean)	110.2 (101.4 to 117)	108.5 (100.5 to 119)	0.94
SAP (LLR)	88.5 (80 to 98)	89 (77 to 100)	0.8
SAP (ULR)	128 (116 to 142)	130 (117 to 143)	0.74
DAP (mean)	54.8 (50.4 to 59.5)	51.5 (46.5 to 56)	0.028
DAP (LLR)	45 (40 to 50)	42 (37 to 46)	0.15
DAP (ULR)	64.5 (57.8 to 69.2)	60 (55 to 66)	0.022
MAP (mean)	73 (69.2 to 79.1)	72 (65.5 to 77)	0.16
MAP (LLR)	61.7 (53 to 65.5)	58 (52 to 65)	0.26
MAP (ULR)	87.5 (81 to 94)	84 (76 to 95)	0.18
CVP (mean)	8.5 (7 to 11.1)	11 (8.5 to 13)	0.00031
CVP (LLR)	4.5 (3 to 6.2)	7 (3 to 8)	0.0042
CVP (ULR)	13 (10 to 16)	15 (12 to 18)	0.00055

^a^AKI−, no or transient acute kidney injury; AKI+, new or persistent acute kidney injury; CO, cardiac output; CVP, central venous pressure; DAP, diastolic arterial blood pressure; LLR, lower limit of the range; MAP, mean arterial blood pressure; SAP, systolic arterial blood pressure; ScvO_2_, central venous oxygen saturation, ULR, upper limit of the range.

Daily fluid balance was calculated as the fluid input (volume of gelatins, crystalloids and feeding) minus fluid output (urine output, fluid from drains and gastric aspiration). Urine was collected upon admission for routine urinary laboratory workup.

### Definition

The diagnosis of AKI was based on the Acute Kidney Injury Network (AKIN) classification. AKI upon admission was defined as an increase in serum creatinine level >50% from baseline or ≥26 μmol/L or oliguria (urinary output <0.5 ml/kg/h for 6 hours). Baseline serum creatinine levels were measured in blood samples taken before hospital admission when available (*n* = 37 (27%)). In cases where the baseline creatinine level or glomerular filtration rate (GFR) was not available, the lowest serum creatinine level measured during the patient’s hospital stay was used if the GFR was ≥75 ml/min/1.73 m^2^ (*n* = 42 (31%)). In other cases, the baseline creatinine level was estimated by using the Modification of Diet in Renal Disease equation with a normal GFR value of 75 ml/min/1.73 m^2^ (*n* = 58 (42%)) [13]. The primary endpoint was the development of a new AKI or persistent AKI during the 5 days following admission. New AKI was defined as (1) an increase in serum creatinine level ≥26 μmol/L or >50% compared to baseline value or (2) need for renal replacement therapy (RRT) after the first 24 hours from admission in patients who had no AKI upon admission. Persistent AKI was defined as a steady or increase in AKIN classification stage between the first 24 hours following admission and day 5 in patients with AKIN stage ≥1 at the time of inclusion in the study. Transient AKI was defined as downstaging of AKI between the first 24 hours following admission and day 5 (for example, from AKIN stage 1 to stage 0). Patients with no AKI or transient AKI are referred to throughout the article as the AKI − group, and patients with new or persistent AKI constitute the AKI + group.

### Statistical analysis

Quantitative parameters are reported as median and interquartile range (IQR; 25th to 75th percentile), and qualitative parameters are expressed as number and percentage. Categorical variables were compared using the χ^2^ test or Fisher’s exact test as appropriate. Continuous variables were compared using the Mann–Whitney *U* test.

#### Primary endpoint

The primary endpoint of the study was to evaluate the AKI + group during the first 5 days after admission with hemodynamic parameters recorded during the first 24 hours after admission. We performed logistic regression analysis, without and with adjustment for potential confounding factors (fluid balance during the first day and average positive end-expiratory pressure (PEEP) level during the first day), to assess the association between CVP level and risk of AKI.

#### Secondary endpoint

The secondary endpoint of the study was the association of AKI with in-hospital mortality, length of stay in the ICU or time from diagnosis of septic shock until death, discharge or transfer. Being alive at discharge was considered a competing event with all-cause in-hospital mortality. The association between AKI + and in-hospital mortality was estimated using cause*-*specific Cox proportional hazards models. Appropriate methods for censored data were used. *P* < 0.05 was considered statistically significant. All analyses were performed using R 2.6.2 statistical software (The R Foundation for Statistical Computing, Vienna, Austria).

## Results

### Patients’ characteristics

The study patients’ characteristics are presented in Table 1. After screening and application of selection criteria, 137 patients were included (Figure 1). AKI was diagnosed in 105 patients (77%) upon ICU admission. From among those patients, 69 were found to have new or persistent AKI after admission. Respectively, 5 (16%) of 32 patients with AKIN stage 1 AKI upon admission, 14 (46%) of 30 patients with AKIN stage 2 AKI upon admission and 35 (47%) of 47 of patients with AKIN stage 3 AKI were subsequently classified as AKI + (that is, persistent AKI).

Thirty-two patients required RRT, which was initiated early (1 day (1 to 2) after ICU admission). The AKI + group scored higher on the Simplified Acute Physiology Score II, as well as higher base deficit and bilirubin levels upon admission. AKI + patients had a higher positive fluid balance during the first 24 hours after admission (3,591.5 ml/kg/h (2,597.5 to 5,714) vs. 2,905 ml/kg/h (1350 to 4717.5); *P* = 0.008) and lower urinary output (0.6 ml/kg/h (0.4 to 1.2) vs. 0.9 ml/kg/h (0.7 to 1.4); *P* = 0.0045). Need for mechanical ventilation, use of vasopressors and/or use of inotropes did not differ between groups. The origin of infection and causative pathogens did not differ between groups either (Table 1).

### Relation between acute kidney injury and systemic hemodynamics

**Figure 2:**
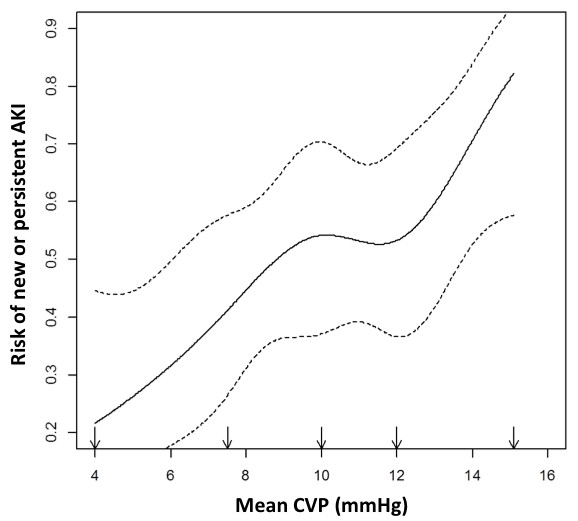
**Statistical model of nonparametric logistic regression showing the relationship between mean central venous pressure during the first 24 hours after admission and the probability of new or persistent acute kidney injury.** Note the plateau for the incidence of acute kidney injury (AKI) when the lower limit of central venous pressure (CVP) was between 8 and 12 mmHg. Over this limit, the rise in CVP was associated with a sharp increase in new or persistent AKI incidence.

The tested hemodynamic variables are presented in Table 2. Only CVP level and DAP (mean and ULR) were statistically different between patients with AKI + and AKI−. CVP values were higher in the AKI + group (4 mmHg (2 to 6) vs. 6 mmHg values in brackets are Interquartile range, as specified in the Methods (statistical analysis) section (3 to 8), respectively; *P* < 0.0001). In addition, CVP was associated with new or persistent AKI (odds ratio (OR) = 1.23 (1.10 to 1.38); *P* = 0.0003). In the full adjusted model, the ORs were 1.05 (0.93 to 1.19; (*P* = 0.3988) for PEEP (for 1 cmH_2_O) and 1.05 (1.01 to 1.09; *P* = 0.0154) for positive fluid balance (for each 250 ml). The association between CVP and new or persistent AKI remained (OR = 1.22 (1.08 to 1.39) for an increase of 1 mmHg; *P* = 0.002) after adjustment for fluid balance and PEEP level), together with a quasi-linear relationship between CVP level and the risk of developing new or persistent AKI (Figure 2). The excretion fraction of sodium was higher (1% (0.3 to 2.9) vs. 0.5% (0.2 to 0.9); *P* = 0.031), and the urine/plasma creatinine ratio (38.3 (23.7 to 62.5) vs. 65.5 (44.1 to 115.3); *P* = 0.001) was lower, in the AKI + group than in the AKI − group. The excretion fraction of urea (26.2% (13.8 to 62.5) vs. 30.1% (18 to 46.5); *P* = 0.21), urinary sodium/potassium ratio (0.6 (0.4 to 1.3) vs. 0.7 (0.4 to 1.3); *P* = 0.77) and plasma urea/creatinine ratio (96.8 (60.9 to 119.6) vs. 100.6 (74.2 to 132.5); *P* = 0.19) did not differ between groups.

### Outcomes

**Figure 3:**
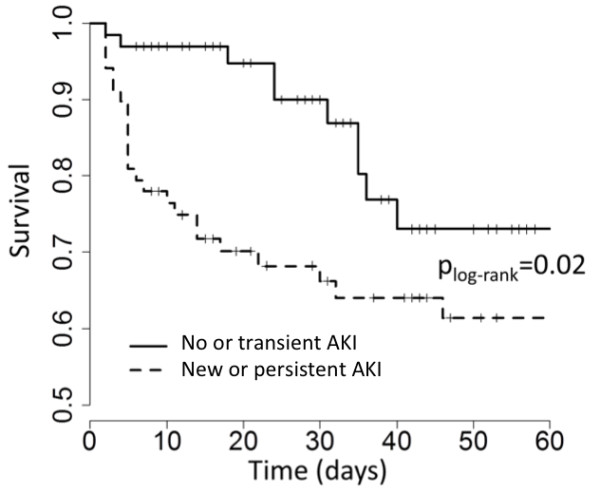
**Survival according to the occurrence of new or persistent acute kidney injury in survival according to the occurrence of AKI.** AKI, acute kidney injury.

The cohort ICU length of stay was 9 days (5 to 17). The mortality rates were 23% (32 patients) and 26% (37 patients) in the ICU and at 28 days, respectively. The AKI + group had a higher mortality rate in the ICU (39% vs. 6%; *P* = 0.0003), in the hospital (45% vs. 16%; *P* = 0.0004) and at day 28 (38% vs. 15%; *P* = 0.003) than AKI − patients (Figure 3). Among the 14 survivors requiring RRT, 1 was continued on it after ICU discharge.

## Discussion

In the present study, we observed a weak association between systemic hemodynamic parameters and the development of AKI among septic ICU patients. The hemodynamic parameter most associated with the development or progression of AKI, regardless of the level of fluid balance and PEEP, was the CVP level. This suggests participation of venous congestion in the physiopathology of AKI in severe sepsis and septic shock.

Although the role of renal hypoperfusion is believed to contribute to the development of sepsis-induced renal dysfunction, AKI appears to be only partially reversible after optimization of systemic hemodynamics [14]. Recently, Schnell *et al*. found that fluid loading did not influence the Doppler renal resistive index in septic ICU patients [6]. Although resuscitation targeting the forward determinants of renal blood flow (MAP and CO) is common, little is known about backward determinants (renal venous pressure). In experimental studies, renal venous congestion has been shown to be important in promoting renal injury [8,15,16]. Uncoupling CO and AKI has also been observed in experimental septic models [17,18]. In patients with acute heart failure, the increase in CVP, but not CO, has been found to be strongly associated with AKI [19,20]. An association between AKI and elevated atrial and brain natriuretic peptide levels in chronic heart failure or after cardiac surgery suggests that cardiac chamber distension by fluid overload and high filling pressure is involved [21]. In the present study, arterial pressure and CO were not statistically different between the two groups of patients, except for DAP. Because renal vascular resistance is low, as evidenced by a positive diastolic blood flow velocity, diastolic perfusion pressure might be a key determinant of renal perfusion [2,6]. The reduction in diastolic flow may result from a decrease in diastolic perfusion pressure related to an increase in renal venous back pressure and/or a decline in DAP [22].

The intravascular level of CVP depends on the patient’s volemic status, right and left heart function, surrounding venous pressure increased by mechanical ventilation, and/or reduced venous compliance. While a patient is in supine position, the renal venous flow depends on renal venous pressure, which is higher than CVP by at least 2 mmHg [23]. The recommended Surviving Sepsis Campaign (SSC) guidelines targeting CVP between “8 and 12 mmHg in spontaneous breathing” or “between 12 and 15 mmHg in patients receiving mechanical ventilation” [10,12] might correspond to a level of renal venous pressure as high as 17 mmHg. To illustrate such a concept, the computed average renal diastolic perfusion pressure was estimated to be approximately 35 mmHg in the AKI + group vs. about 42 mmHg in the AKI − group, a difference that may affect glomerular filtration pressure.

Fluid resuscitation and pressure optimization to better perfuse the kidney, a landmark treatment for septic patients, is based on the improvement of renal perfusion pressure. For some patients, the induced CVP elevation may overcome the DAP increase, reducing renal perfusion with harmful effects on renal function. This aspect is supported by the recently reported association between fluid overload and mortality in critically ill patients, especially in patients with AKI or septic shock [24], and confirmed by *post hoc* analysis of the Vasopressin and Septic Shock Trial [25]. The investigators in that trial reported that a positive fluid balance and elevated CVP were associated with increased mortality in patients with septic shock [25]. Other factors may also accompany the venous congestion mechanism, such as an increase in renal interstitial pressure associated with hyperpermeability and inflammatory cell adherence [26-28]. The creation of a vicious circle with oliguria and fluid-loading may then aggravate AKI. Therefore, achieving a defined CVP as a therapeutic target might not be suitable in septic patients. Our study suggests instead that hemodynamic targets are best achieved at low CVP (that is, CVP less than 8 to 12 mmHg). The SSC guidelines mention that “in mechanically ventilated patients or those with known preexisting decreased ventricular compliance, a higher target CVP of 12–15 mmHg should be achieved to account for the impediment in filling” [12]. The vast majority (86%) of our patients were under mechanical ventilation during the first 24 hours after ICU admission and would therefore be expected to achieve higher CVP. The results of our study suggest, however, that such targets might be too high from a renal perspective. The strategy to be applied in patients presenting with high CVP or with elevation of CVP during resuscitation requires additional studies, but fluid restriction in these patients is an important option to be considered.

Our study has several limitations. First, the sample size was rather small, thus our results must be confirmed in a larger, multicenter cohort. Only patients with hemodynamic monitoring, including CVP, were included, which may have introduced bias. In this respect, we selected patients with the most severe forms of sepsis, as defined by the high Sequential Organ Failure Assessment scores, with the large majority of them being under mechanical ventilation and treated with vasopressors. Also, a high proportion (32%) of patients received RRT, which appears to be consistent with a recently published observational cohort of patients with septic shock [29].

Second, the study design was noninterventional, and the association between CVP and AKI does not prove a causal relationship. Whether actively decreasing CVP may improve outcomes and prevent AKI needs further evaluation, and which strategy should be applied to patients with high CVP merits further study.

## Conclusions

In this study, we observed a loose association between most systemic hemodynamic parameters and development of new or persistent AKI in septic patients, with the exception of CVP. The association of the level of CVP and the risk of developing AKI suggests a role of venous congestion in the development of AKI. The paradigm targeting high CVP to reduce occurrence of AKI should be reconsidered in this setting.

## Key messages

•AKI progresses in about 50% of septic patients despite hemodynamic optimization.

•We observed a weak association between systemic hemodynamic parameters and AKI in septic patients.

•Higher mean CVP in the first 24 hours was linearly associated with increasing risk of new or persistent AKI across all observed CVP values.

•The association of elevated CVP with AKI suggests a role of venous congestion in the development of AKI.

•The paradigm that targeting high CVP may reduce the occurrence of AKI should be revised.

## Abbreviations

AKI: Acute kidney injury; CO: Cardiac output; CVP: Central venous pressure; DAP: Diastolic arterial blood pressure; GFR: Glomerular filtration rate; LLR: Lower limit range; MAP: Mean arterial blood pressure; PEEP: Positive end-expiratory pressure; ScvO2: Central venous oxygen saturation; ULR: Upper limit of the range.

## Competing interests

The authors declare that they have no competing interests.

## Authors’ contributions

ML and DP conceived and designed the study, contributed to the analysis and interpretation of data as well as drafting the manuscript, and gave their final approval of the version to be published. CD, CS, JM, EG and ACL contributed to the acquisition, analysis and interpretation of data and gave their final approval of the version to be published. All authors read and approved the final manuscript.
